# Moscatilin alleviates oxidative stress and inflammatory response of Müller cells in diabetic retinopathy through suppressing the p38 mitogen‐activated protein kinase/c‐Jun N‐terminal kinase and nuclear factor kappa‐B signaling pathways

**DOI:** 10.1002/ccs3.12059

**Published:** 2025-02-19

**Authors:** Suhua Zhu, Man Zhang, Zhen Qu, Shengqiu Xu, Jie Peng, Fanjing Jiang

**Affiliations:** ^1^ Department of Pharmacy Xuzhou No.1 People's Hospital Xuzhou Jiangsu China; ^2^ Department of Pharmacy Yancheng No.1 People's Hospital Yancheng Jiangsu China

**Keywords:** diabetic retinopathy, moscatilin, Müller cells, NF‐κB, p38 MAPK/JNK

## Abstract

Diabetic retinopathy (DR), as the main ophthalmic complication of diabetes mellitus, is a major eye disorder contributing to blindness. Oxidative stress and inflammation in retinal Müller cells participate in the pathogenesis of DR. This work aims to study the biological role of moscatilin in the progression of DR and the underlying mechanism. High glucose (HG)‐stimulated mouse primary retinal Müller cells and high‐fat diet + streptozotocin (STZ)‐induced DR mouse models were constructed as in vitro and in vivo models, respectively. The effects of moscatilin treatment on oxidative stress and inflammation in HG‐stimulated Müller cells and DR mice were evaluated by detecting intracellular reactive oxygen species production, malondialdehyde levels, superoxide dismutase and catalase activities, glutathione/oxidized glutathione ratio, as well as proinflammatory cytokine levels through CM‐H_2_DCFDA staining, commercial kits, and enzyme‐linked immunosorbent assay. Dual immunofluorescence staining of glial fibrillary acidic protein and vimentin was used to evaluate the development of Müller cells in mouse retinas. The activity of p38 mitogen‐activated protein kinase (MAPK)/c‐Jun N‐terminal kinase (JNK) and nuclear factor kappa‐B (NF‐κB) signaling pathway was assessed through western blotting and immunofluorescence staining. Moscatilin pretreatment prevented HG‐induced decrease in Müller cell viability. Moscatilin mitigated oxidative stress, inflammation, and extracellular matrix remodeling in HG‐stimulated Müller cells and DR mice. Mechanically, moscatilin reduced the levels of receptor for advanced glycation end products, phosphorylated I‐kappa‐B‐alpha, p‐p65 NF‐κB, p‐p38 MAPK, and p‐JNK in both HG‐stimulated Müller cells and DR mice. Moscatilin plays an antioxidant and anti‐inflammatory role in DR by inhibiting the p38 MAPK/JNK and NF‐κB signaling pathways.

## Graphical abstract

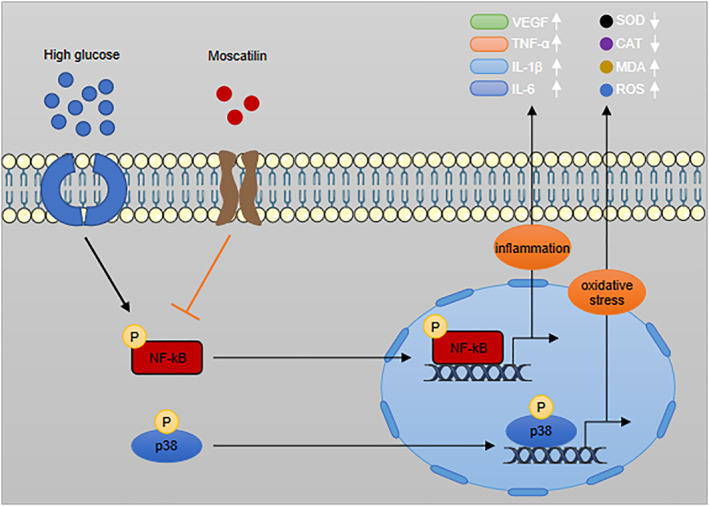

## 1 INTRODUCTION

Diabetes mellitus (DM) is a metabolic disease characterized by chronic hyperglycemia due to multiple etiologies and is caused by defective insulin secretion and/or action.^1^ DM is one of the most common chronic diseases and has become a worldwide public health problem that poses a serious threat to human health.^2^ Epidemiological studies have shown that DM is most prevalent in middle‐aged and older adults, with a cumulative number of cases exceeding 300 million worldwide.^3^ Although diabetes itself is not life‐threatening, long‐standing hyperglycemia can lead to chronic damage and dysfunction of various tissues and organs, especially the eyes, kidneys, heart, blood vessels, and nerves, and induce a series of acute and chronic complications, all of which can be life‐threatening.^4^ Diabetic retinopathy (DR), as one of the most serious and common diabetic microvascular complications, has been recognized as one of the foremost ocular diseases causing blindness.^5^ It was reported that the blindness rate of DR is nearly 25 times higher than that of the nondiabetic blind population, which is the highest rate of double blindness.^6^ Generally, DR exhibits no obvious symptoms in its early stages, but patients will experience symptoms such as flying mosquitoes, visual field defects, and blurred vision until it progresses to an advanced stage, which can eventually result in irreversible loss of vision.^7^ Nevertheless, the pathogenesis of DR has not been fully elucidated up‐to‐date, and this lacks effective early diagnostic and therapeutic methods for DR.

Previous studies on the pathogenesis of DR mainly focused on vascular lesions, but recent studies have shown that retinal neurodegeneration, including degeneration and loss of retinal neurons and glial cells, precedes the development of retinal vascular lesions in DR.^8^ Müller cells, as the most important glial cells in the retina, are distributed throughout the entire retina, encircling the cytosol, protuberances, and retinal vessels of neurons at all levels of the retina.^9^ Müller cells are the bridge between blood vessels and nerve cells in the retina, which not only support and nourish neurons, but also maintain the stability of extracellular ions, participate in the glutamate cycle and synaptic transmission, and even have an important influence on the primary integration and processing of visual signals.^10^ Müller cells can not only affect the retinal vascular lesions, but also affect the retinal nerve cell lesions, so they inevitably become one of the main target cells in DR research.^11^ Glutamate is the main neurotransmitter in the retina. It has been found that the concentration of glutamate in the retina increases during DR, and the high concentration of glutamate causes the opening of Ca^2+^ channels in the retinal cells, which leads to intracellular Ca^2+^ overload and initiates a cascade reaction and causes the death of retinal cells.^12^ Müller cells are the only retinal cells that can synthesize glutamine synthetase (GS), which converts glutamate to glutamine and removes excess extracellular glutamate.^13^ Therefore, Müller cells play a critical role in maintaining the balance of glutamate in the retina and maintaining normal retinal function.^14^ In the early stage of DR, Müller cells become dysfunctional, and their ability to remove glutamate decreases, leading to the accumulation of glutamate and corresponding neuronal function decrease, loss, or even death.^15^ In addition, high blood glucose can cause the enhancement of oxidative stress and the generation of more reactive oxygen species (ROS), which further promotes the expression of various inflammatory factors, leading to Müller cell dysfunction and even accelerating apoptosis.^16^ Accordingly, suppressing oxidative stress and inflammatory responses in Müller cells in a hyperglycemic environment may ameliorate retinal vascular and neural damage, thereby mitigating the progression of DR.

Currently, laser and anti‐vascular endothelial growth factor (VEGF) therapies, such as intravitreal injection of bevacizumab and ranibizumab, are the major therapeutic strategies for DR, which however, are unacceptable to most patients owing to their long treatment duration, obvious side effects, and high cost.^17^, ^18^ Hence, it is urgently required to discover alternative drugs and therapies for DR. Traditional Chinese medicine (TCM) is a critical part of world medicine, which has formed a unique system and exerted a curative effect for thousands of years.^19^ Chinese herbal medicines, with the advantages of easy access, fewer side effects, lower price, and good curative effects, are widely used in China. TCM and its prescriptions, which can clear away heat and fire, activate blood and remove blood stasis, and replenish Qi, are frequently employed to improve DR‐related symptoms.^20^ In recent years, expanding monomer compounds have been extracted from TCM to treat DR.^21^ Moscatilin (the chemical structure is shown in Figure 2A) is a bibenzyl derivative extracted from the orchid, *Dendrobium moscatum*.^22^ It has been well‐documented that moscatilin possesses pharmacological capabilities including antiplatelet aggregation, anticancer, anti‐inflammatory, and antioxidant effects.^23^ Moscatilin was previously reported to alleviate SH‐SY5Y neuronal cell injury induced by advanced glycation end products through the activation of the adenosine 5‘‐monophosphate‐activated protein kinase signaling pathway, showing its therapeutic potential in diabetic/hyperglycemia‐associated neurodegenerative diseases.^24^ Furthermore, Xu et al. demonstrated that *Dendrobium*, the source of moscatilin, had potential values in the clinical management of type 2 diabetes^25^ Chao et al. suggested that hypoxia/ischemia‐triggered damage in retinal cells could be mitigated by moscatilin at concentrations equal to or less than 1 μmol/L.^26^ However, the functions and molecular mechanisms of moscatilin in DR development remain elusive.

In this study, high glucose (HG)‐stimulated Müller cells and high‐fat diet + streptozotocin (STZ)‐induced DR mouse models were used as cellular and animal models to investigate the role of moscatilin in the progression of DR and confirm whether it exerts a protective role in DR by modulating the nuclear factor kappa‐B (NF‐κB) and p38 mitogen‐activated protein kinase (MAPK)/c‐Jun N‐terminal kinase (JNK) pathways.

## 2 MATERIALS AND METHODS

### 2.1 Cell isolation, culture, and identification

Mouse primary retinal Müller cells were isolated based on the methods described in the previous report ^27^ of 5‐ to 7‐day‐old newborn C57BL/6 pups, which were purchased from Jackson Laboratories (Bar Harbor, ME). In brief, eyeballs were removed after the mice were sacrificed by cervical dislocation and then washed with phosphate‐buffered saline (PBS). Then, the retinas were peeled off from the eyeballs under sterile conditions, minced into 1 mm^3^ pieces, and digested with trypsin for 5 min. The mixture of cells and trypsin were mixed fully with Dulbecco's Modified Eagle's Medium (DMEM; Thermo Fisher Scientific, Waltham, MA, USA) supplemented with 10% fetal bovine serum (FBS; Gibco, Grand Island, NY, USA), 100 mg/mL streptomycin, and 100  U/mL penicillin after being moved into a sterile centrifuge tube. The mixture was filtrated with a stainless steel sieve and centrifuged to collect the cell pellets, which were then resuspended in complete DMEM and cultured at 37°C with 5% CO_2_ in the cell culture flask (Corning, New York, USA). The medium was replaced every 2 days. The cells were subcultured after cell fusion. The 2‐3 passages of Müller cells were used in follow‐up in vitro experiments. The morphology of Müller cells was observed under a phase contrast microscope. Moreover, fluorescence staining for GS was performed for cell identification, and fluorescent cells were visualized with a fluorescence microscope (Leica, Germany).

### 2.2 Cell treatment

Moscatilin powder (purity: 98%; Cat. No. BCP48233; Shanghai Biochempartner Co., Ltd., Shanghai, China) was dissolved in dimethyl sulfoxide (DMSO) (purity: 99.8%; Cat. No. 925578; Shanghai Zhengyu Biotechnology Co., Ltd., Shanghai, China) to create a stock at a concentration of 100 μmol/L and subsequently diluted in a culture medium to appropriate concentrations (0.1, 0.5, and 1 μmol/L) for follow‐up experiments. DMSO was utilized as the vehicle control. The final concentration of DMSO was less than 0.1% (v/v), a concentration that did not influence cell viability.^28^ To induce cell injury, Müller cells were exposed to normal (5.5 mmol/L) or high (15, 25, 35, 45, and 55 mmol/L) D‐glucose for 24 h or to high (35 mmol/L) D‐glucose for 6, 12, 24, 48, and 96 h. To assess the influence of moscatilin on high glucose (HG)‐induced cell injury, Müller cells were pretreated with moscatilin (0.1, 0.5, and 1 μmol/L) for 24 h and were subsequently stimulated by high (35 mmol/L) D‐glucose for 24 h.

### 2.3 CCK‐8 assay

The viability of Müller cells was measured by using a Cell Counting Kit‐8 (CCK‐8; Cat. No. C6005; New Cell & Molecular Biotech Co., Ltd., Suzhou, China) following the manufacturer's instructions. In short, cells were seeded at a density of 5 × 10^3^ cells/well in 96‐well plates. After 24 h, the cells received the above‐mentioned treatment and were incubated at 37°C with 5% CO_2_. At the end of the 24‐h incubation period, cells were washed with Hank's solution (Cat. No. ZY91105; Shanghai Zeye Biotechnology Co., Ltd., Shanghai, China), followed by the addition of a 100‐μL medium containing 10 μL of CCK‐8 solution into each well. Cells were allowed a further 2‐h culture in a CO_2_ incubator at 37°C, and the absorbance at 450 nm was measured by a microplate reader (SpectraMax M5, Molecular Devices, USA).

### 2.4 Animals

The animal experiments were reviewed and approved by the Institutional Ethics Review Committee of Xuzhou No.1 People's Hospital. Thirty‐two 8‐week‐old male C57BL/6J mice purchased from Beijing Beiyou Biotechnology Co., Ltd (Beijing, China) were housed in a controlled environment (humidity 55%–60%, a 12‐h light‐dark cycle, and 21–23°C) with unlimited access to standard water and food. The mice were randomized into sham, DR, DR + DMSO, and DR + Moscatilin groups (*n* = 8 per group). The induction of diabetes in mice was performed as previously described.^29^ Mice were fasted overnight and then daily fed with a high‐fat diet (Cat. No. D12492; Research Diets Inc., New Brunswick, NY, USA) and intraperitoneally injected with 50 mg streptozotocin (STZ; Cat. No. M5721; Nanjing Wobo Biotechnology Co., Ltd.; Nanjing, China) for 5 consecutive days. Mice in the sham group were raised with a normal diet and intraperitoneally injected with normal saline every day. Mice with a blood glucose level higher than 16.7 mmol/L were considered as diabetic and used in subsequent experiments.^16^ Moscatilin was dissolved in DMSO to create a 50 mg/mL stock concentration. After 6 weeks, the diabetic mice were administered through intraperitoneal injection with moscatilin (25 mg/kg) or equal amounts of vehicle daily for 7 days.^30^ Finally, the mice were euthanized by cervical dislocation and their retinal tissues were dissected for further analysis.

### 2.5 Enzyme‐linked immunosorbent assay

Müller cells after the indicated treatment were harvested and dissociated in lysis buffer to obtain the cell lysates, and the supernatants were collected by centrifugation for 10 min at 20,000 rpm. The equiponderant mouse retinal tissues were dissociated in radio‐immunoprecipitation assay (RIPA) lysis buffer (Cat. No. abs9229; Absin, Shanghai, China) with protease inhibitors (MedChemExpress, USA). The homogenates were centrifuged for 15 min at 12,000 rpm at 4°C to obtain the supernatants. The levels of VEGF (Cat. No. EMC103), tumor necrosis factor‐α (TNF‐α; Cat. No. NBS‐EMC102a.96), interleukin‐6 (IL‐6; Cat. No. EMC004(H).96), and interleukin‐1β (IL‐1β; Cat. No. EMC001b.96) in the cellular supernatant and retina were detected using commercial enzyme‐linked immunosorbent assay (ELISA) kits (Neobioscience, Shenzhen, China) in relative to the manufacturer's instructions. By measuring the absorbance of each microwell at 450 nm using a NanoDrop 2000 spectrophotometer (Thermo Scientific, Wilmington, DE, USA), the concentrations of these cytokines were quantified according to the standard curve.

### 2.6 Examination of oxidative stress markers

The level of malondialdehyde (MDA; Cat. No. HZ0131M; Shanghai Huzhen Industrial Co., Ltd., Shanghai, China), activities of superoxide dismutase (SOD; Cat. No. 50101ES01; Yeasen Biotechnology Co., Ltd., Shanghai, China) and catalase (CAT; Cat. No. 50108ES60; Yeasen Biotechnology Co., Ltd.), and the ratio of glutathione/oxidized glutathione (GSH/GSSG; Cat. No. HZ0053; Shanghai Huzhen Industrial Co., Ltd.) in supernatants of cultured Müller cells and homogenates of mouse retinal tissues were examined using the corresponding assay kits. The absorbance was recorded at 532 nm (for MDA), 450 nm (for SOD), 520 nm (for CAT), and 405 nm (for GSH/GSSG) using a microplate reader.

### 2.7 Detection of ROS levels

The fluorescence probe 5‐(and‐6)‐chloromethyl‐2′,7′‐dichlorodihydrofluorescein diacetate (CM‐H_2_DCFDA; Cat. No. 850013‐49‐9; MedChemExpress) was used to estimate the intracellular production of ROS. Briefly, Müller cells were grown in 24‐multiwell plates and received indicated treatments. Fresh mouse retinal tissues were harvested and processed to prepare frozen sections. Thereafter, cells and tissue sections were incubated for 30 min at 37°C with 10 μM CM‐H_2_DCFDA, followed by counterstaining for 10 min at room temperature with 4,6‐Diamidino‐2‐phenylindole (DAPI; Cat. No. KGA215; Shanghai Zhennuo Biological Technology Co., Ltd., Shanghai, China). The fluorescence intensity was evaluated with a fluorescence microplate reader at the excitation and emission wavelengths of 488 and 525 nm, respectively.

### 2.8 Western blotting

Total protein from Müller cells or mouse retinal tissues was extracted using RIPA lysis buffer with protease inhibitors following the manufacturer's protocols. After centrifugation, the supernatant was harvested to determine the protein concentration by the bicinchoninic acid method. Then, an equal amount (25 μg per sample) of protein was separated by sodium dodecyl sulfate‐polyacrylamide gel electrophoresis (SDS‐PAGE) and electrotransferred to polyvinylidene fluoride membranes (Millipore, Billerica, MA, USA). The membranes were blocked in skim milk (5%, w/v), followed by incubation with primary antibodies against receptor for advanced glycation end products (RAGE; Cat. No.ab216329; 1:1000; Abcam, Shnaghai, China), phosphorylated I‐kappa‐B‐alpha (p‐IkBα; Cat. No. AF2002; 1:1000; Affinity Biosciences, Cincinnati, OH, USA), IkBα (Cat. No. AF7776; 1:1000; Affinity Biosciences), p‐p65 (Cat. No. ab76302; 1:1000; Abcam, Cambridge, UK), p65 (Cat. No. ab32536; 1:1000; Abcam), p‐p38 (Cat. No. AF4001; 1:1000; Affinity Biosciences), p38 (Cat. No. AF6456; 1:1000; Affinity Biosciences), p‐JNK (Cat. No. AF3318; 1:1000; Affinity Biosciences), JNK (Cat. No. AF6318; 1:1000; Affinity Biosciences), matrix metallopeptidase 2 (MMP2; Cat. No. ab92536; 1:1000; Abcam), matrix metallopeptidase 9 (MMP9; Cat. No. ab228402; 1:1000; Abcam), tissue inhibitor of metalloproteinase 2 (TIMP2; Cat. No. ab180630; 1:1000; Abcam), and β‐actin (Cat. No. AF7018; 1:3000; Affinity Biosciences) at 4°C overnight. Afterward, the membranes were washed three times (15 min each time) with Tris‐buffered saline and Tween 20 (TBST; Cat. No. T1082; Solarbio, Beijing, China) and hybridized with horseradish peroxidase‐conjugated secondary antibodies (Abcam) at approximately 25°C for 1 h. The protein bands were visualized using a chemiluminescence substrate (Cat. No. pro_57077; Bio Excellence International Tech Co., Ltd., Beijing, China) and quantified using ImageJ software. The β‐actin band served as the loading control.

### 2.9 Gelatin zymography

To determine the enzymatic activity of MMP2 and MMP9, the conditioned media of cells were collected, centrifuged at 4000 rpm for 10 min at 4°C, and mixed with standard SDS‐gel loading buffer containing 0.01% SDS without boiling or reduction before loading. Prepared samples were loaded onto 8% SDS polyacrylamide gels containing 0.5% gelatin for electrophoresis. To remove SDS, the gels were soaked for 30 min at room temperature in 0.25% Triton X‐100, followed by overnight incubation at 37°C in digestion buffer containing 5 mM phenylmethanesulfonyl fluoride to allow proteinase digestion of its substrate. Subsequently, the gels were stained for 2 h with 0.25% Coomassie brilliant blue R‐250 (Cat. No. C8430; Solarbio) and destained with a destaining solution (70% water, 20% methanol, and 10% acetic acid). Gelatinolytic activity appeared as clear bands of digested gelatin and was analyzed using ImageJ analysis software.

### 2.10 Immunofluorescence staining

Mouse retinal tissues were subject to 1 h fixation under 4°C using 4% paraformaldehyde, followed by gradient dehydration with 20% and 30% sucrose solutions and embedding within the optical coherence tomography compound. Then, the retinal samples were cut into 5 μm slices with a freezing microtome (Leica, Nussloch, Germany), which were rewarmed for 30 min at room temperature, blocked for 1 h with 5% bovine serum albumin under ambient temperature, and incubated overnight with anti‐glial fibrillary acidic protein (GFAP; Cat. No. DF6040; 1:500; Affinity Biosciences) and anti‐vimentin (Cat. No. AF7013; 1:200; Affinity Biosciences) or with anti‐p‐p65 (Cat. No. AF3387; 1:200; Affinity Biosciences) or anti‐p‐p38 (Cat. No. AF4001; 1:200; Affinity Biosciences) primary antibodies at 4°C. The next day, the sections were sufficiently rinsed with PBS, followed by an additional 1 h incubation with fluorescence secondary antibodies Alexa‐Fluor 488 goat anti‐rabbit (Cat. No. S0018; 1:200; Affinity Biosciences) and Alexa‐Fluor 647 goat anti‐rabbit (Cat. No. S0013; 1:200; Affinity Biosciences) under ambient temperature. The slides were washed, counterstained for 10 min in the dark with DAPI, and sealed with the anti‐fade mounting agent. Images were acquired by employing an Olympus FV1000 confocal laser scanning microscope. ImageJ software was used to analyze the fluorescence intensity.

### 2.11 Statistical analysis

All experiments were conducted with at least three biological replicates and all data were analyzed by using Prism 9 (GraphPad Software, CA, USA). Data were confirmed to follow normal distribution by using a Shapiro‐Wilk test. Statistical details were calculated by Student's *t*‐test (two groups) or one‐way analysis of variance followed by Tukey's post hoc test (multiple groups). All experimental data were expressed as mean ± standard deviation. The difference was considered significant if *p* < 0.05.

## 3 RESULTS

### 3.1 Identification of mouse primary retinal Müller cells

**FIGURE 1:**
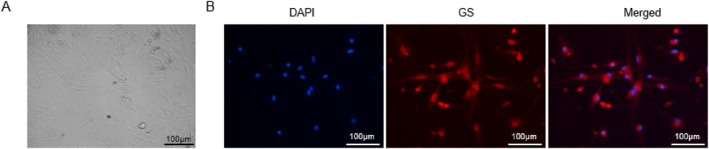
Identification of mouse primary retinal Müller cells. (A) Observation of Müller cell morphology under a phase contrast microscope. Scale bar = 100 μm. (B) Fluorescence staining for Müller cell‐specific enzyme glutamine synthetase. Scale bar = 100 μm.

Under phase contrast microscopy, Müller cells were oval‐shaped and in a pale color with abundant cytoplasm, a big nucleus, and long pyramidal projections at both ends of the body (Figure 1A). As revealed by GS staining, almost all cells were stained with GS, a Müller cell‐specific enzyme in the retina (Figure 1B).

### 3.2 Moscatilin suppresses HG‐induced reduction in the viability of Müller cells

**FIGURE 2:**
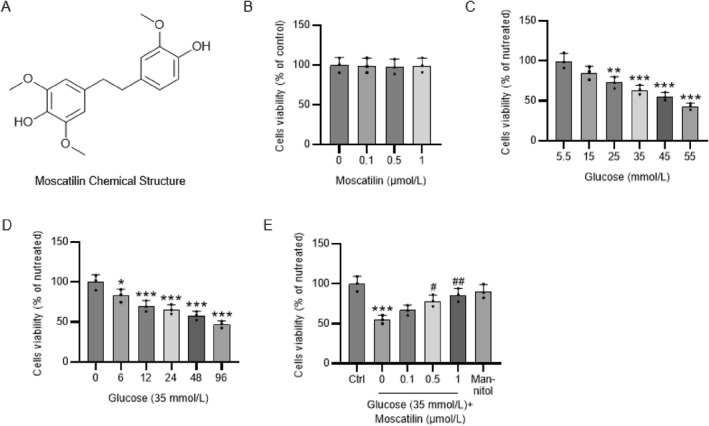
Moscatilin ameliorates HG‐induced reduction in the viability of Müller cells. (A) The chemical structure of moscatilin. (B) CCK‐8 assay of Müller cell viability after 24 h treatment with moscatilin (0.1, 0.5, and 1 μmol/L). (C–D) CCK‐8 assay of Müller cell viability after stimulation by normal glucose (5.5 mmol/L) or HG (15–55 mmol/L) for 24 h or by HG (35 mmol/L) for 6, 12, 24, 48, and 96 h. (E) CCK‐8 assay of Müller cell viability after pretreatment with moscatilin (0.1, 0.5, and 1 μmol/L) for 24 h and stimulation by HG (35 mmol/L) for 24 h. Mannitol (25 mM) was used as osmotic control. *n* = 3. *p < 0.05, **p < 0.01, ***p < 0.001 versus control; #*p* < 0.05, ##*p* < 0.01 versus HG (35 mmol/L). Values are expressed as the mean ± standard deviation of triplicate experiments. HG, high glucose.

First, we discovered that 24 h treatment with moscatilin (0.1, 0.5, and 1 μmol/L) exerted no influence on the viability of Müller cells, suggesting that moscatilin (0.1, 0.5, and 1 μmol/L) had no cytotoxicity on Müller cells (Figure 2B). In contrast, HG stimulation resulted in concentration (15–55 mmol/L)‐dependent reductions in Müller cell viability (Figure 2C). Furthermore, a time‐dependent decrease in Müller cell viability was observed after incubation with HG (35 mmol/L) for 6, 12, 24, 48, and 96 h (Figure 2D). Taken together, HG concentration‐ and time‐dependently induced cell injury in Müller cells. To evaluate the effects of moscatilin on HG‐triggered cell injury, Müller cells were pretreated for 24 with moscatilin (0.1, 0.5, and 1 μmol/L) before HG (35 mmol/L) stimulation. It was shown that HG markedly attenuated Müller cell viability whereas mannitol (25 mM; as osmotic control) didn't affect cell viability. However, moscatilin pretreatment prevented HG‐induced decrease in Müller cell viability (Figure 2E).

### 3.3 Moscatilin mitigates oxidative stress by inhibiting RAGE in HG‐stimulated Müller cells

**FIGURE 3:**
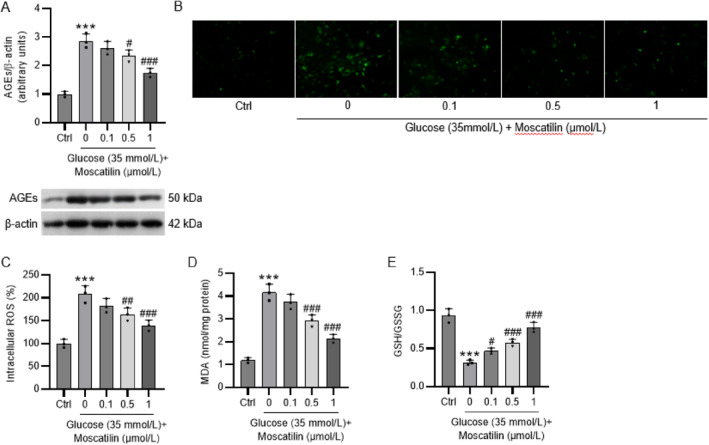
Moscatilin mitigates oxidative stress by inhibiting RAGE in HG‐stimulated Müller cells. Müller cells were pretreated with moscatilin (0.1, 0.5, and 1 μmol/L) for 24 h, followed by stimulation by HG (35 mmol/L) for 24 h. (A) Determination of RAGE protein by western blotting. (B–C) Measurement of intracellular reactive oxygen species production by using the oxidation‐sensitive fluoroprobe CM‐H_2_DCFDA as a substrate. (D) Estimation of lipid peroxidation by detecting MDA levels with the commercial kit. (E) Examination of the GSH/GSSG ratio using the commercial kit. *n* = 3. ***p < 0.001 versus control; #*p* < 0.05, ##*p* < 0.01, ###*p* < 0.001 versus HG (35 mmol/L). Values are expressed as the mean ± standard deviation of triplicate experiments. GSH/GSSG, glutathione/oxidized glutathione; HG, high glucose; MDA, malondialdehyde; RAGE, receptor for advanced glycation end products.

As shown in Figure 3A, HG stimulation resulted in a remarkable increment in the RAGE protein level in Müller cells, while moscatilin pretreatment reduced the accumulation of RAGE in a concentration‐dependent manner in HG‐exposed Müller cells. The generation of ROS in Müller cells was significantly enhanced after HG exposure, which however, was inhibited by moscatilin pretreatment (Figure 3B,C). Besides, the intracellular MDA levels in Müller cells incubated with HG were notably higher than in control cells, while pretreatment with moscatilin effectively suppressed HG‐induced elevation in MDA levels in Müller cells (Figure 3D). The decline in the GSH/GSSG ratio caused by HG exposure in Müller cells was reversed by moscatilin pretreatment in a concentration‐dependent manner (Figure 3E).

### 3.4 Moscatilin alleviates inflammation by inhibiting NF‐κB signaling in HG‐stimulated Müller cells

**FIGURE 4:**
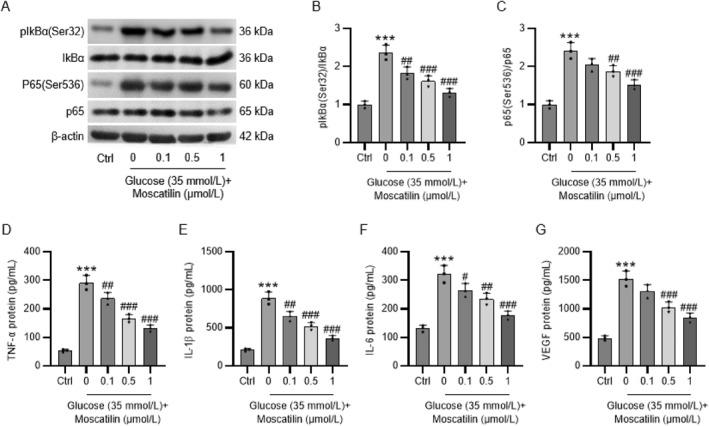
Moscatilin alleviates inflammation by inhibiting NF‐κB signaling in HG‐stimulated Müller cells. Müller cells were pretreated with moscatilin (0.1, 0.5, and 1 μmol/L) for 24 h, followed by exposure to HG (35 mmol/L) for 24 h. (A–C) Western blotting of p‐IkBα, IkBα, p‐p65, and p65 protein levels in Müller cells. (D–G) ELISA of TNF‐α, IL‐1β, IL‐6, and VEGF levels in Müller cells. *n* = 3. ***p < 0.001 versus control; #*p* < 0.05, ##*p* < 0.01, ###*p* < 0.001 versus HG (35 mmol/L). Values are expressed as the mean ± standard deviation of triplicate experiments. ELISA, enzyme‐linked immunosorbent assay; HG, high glucose; IL‐1β, interleukin‐1β; IL‐6, interleukin‐6; p‐IkBα, phosphorylated I‐kappa‐B‐alpha; TNF‐α, tumor necrosis factor‐α; VEGF, vascular endothelial growth factor.

Next, whether moscatilin affects inflammation, which contributes to the pathogenesis of DR, in HG‐exposed Müller cells was explored. Before measuring the levels of inflammatory cytokines, we assessed the expression of proteins related to NF‐κB signaling pathway, which plays a crucial role in triggering inflammatory response. Western blotting revealed that p‐IkBα and p‐p65 protein levels in Müller cells were evidently upregulated after HG exposure, while moscatilin pretreatment suppressed HG‐induced elevation in p‐IkBα and p‐p65 protein levels, suggesting that moscatilin inhibited NF‐κB pathway in HG‐stimulated Müller cells (Figure 4A–C). Subsequently, we observed through the data of ELISA that TNF‐α, IL‐1β, IL‐6, and VEGF levels were considerably higher in HG‐stimulated Müller cells than in control Müller cells, but were lower in moscatilin‐pretreated and HG‐stimulated Müller cells than in HG‐stimulated Müller cells (Figure 4D–G).

### 3.5 Moscatilin

Ameliorates extracellular matrix remodeling by suppressing p38/JNK signaling in HG‐exposed Müller cells.

**FIGURE 5:**
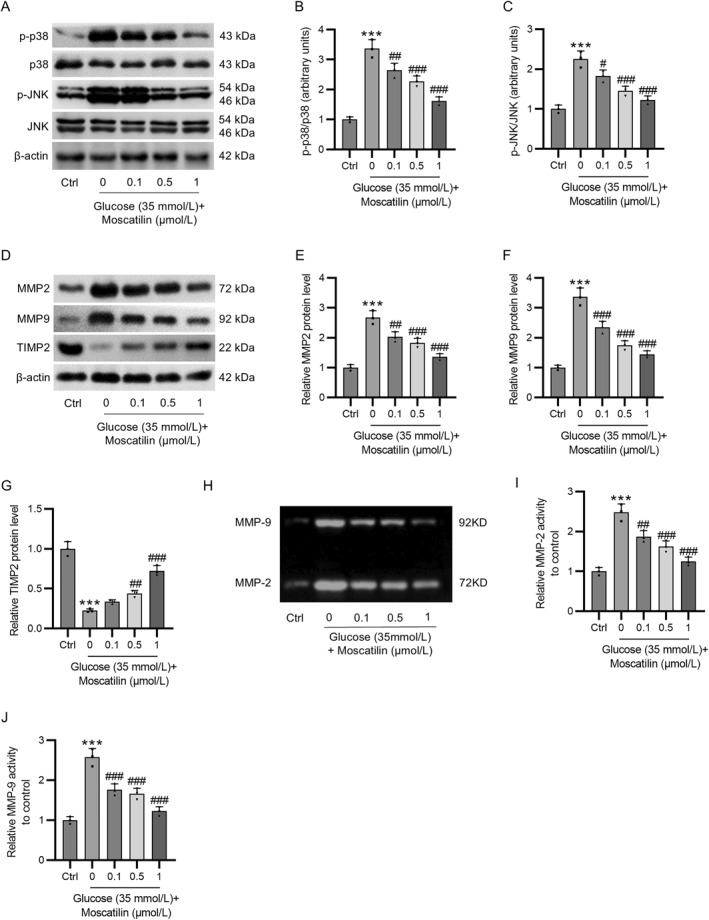
Moscatilin ameliorates extracellular matrix remodeling by suppressing p38/JNK signaling in HG‐exposed Müller cells. Müller cells were pretreated with moscatilin (0.1, 0.5, and 1 μmol/L) for 24 h, followed by exposure to HG (35 mmol/L) for 24 h. (A–C) Western blotting of p‐p38, p38, JNK, and p‐JNK protein levels in Müller cells. (D–G) Western blotting of MMP2, MMP9, and TIMP2 protein levels in Müller cells. (H–J) Determination of MMP2 and MMP9 activity by gelatin zymography in Müller cells. *n* = 3. ***p < 0.001 versus control; #*p* < 0.05, ##*p* < 0.01, ###*p* < 0.001 versus HG (35 mmol/L). Values are expressed as the mean ± standard deviation of triplicate experiments. HG, high glucose; JNK, c‐Jun N‐terminal kinase; MMP2, matrix metallopeptidase 2; MMP9, matrix metallopeptidase 9; TIMP2, tissue inhibitor of metalloproteinase 2.

The remodeling of extracellular matrix (ECM) components is associated with basement membrane thickening in DR, which can be regulated by the p38/JNK signaling pathway. As revealed by western blotting, HG stimulation led to a marked increment in p‐p38 and p‐JNK protein levels in Müller cells. Nevertheless, such an increase was repressed by pretreatment with moscatilin (Figure 5A–C), which suggested that moscatilin counteracted HG‐induced p38/JNK pathway activation in Müller cells. In addition, the levels of matrix metalloproteinases (MMPs), MMP2 and MMP9, which contribute to the degradation of key ECM components, as well as the level of tissue inhibitor of MMP2 (TIMP2) were examined in Müller cells. We discovered that moscatilin inhibited HG‐induced elevation in MMP2 and MMP9 protein levels and reduction in TIMP2 protein levels in a concentration‐dependent manner (Figure 5D–G). In the meantime, HG‐induced enhancement in the activity of MMP2 and MMP9 was concentration‐dependently suppressed by moscatilin pretreatment (Figure 5H–J).

### 3.6 Moscatilin attenuates oxidative stress and inflammation in DR mouse models

To provide the in vivo evidence to further validate the therapeutic effects of moscatilin in DR, the mouse models of DR were constructed and the levels of oxidative stress and inflammation markers were detected. Since a high glucose environment can lead to Müller cell apoptosis and loss and thereby result in retinal dysfunction, we performed dual‐immunofluorescence staining by using anti‐GFAP and anti‐vimentin antibodies on mouse retinal sections to evaluate the development of Müller cells. We observed that the fluorescence intensity of both GFAP and vimentin was prominently lower in the DR group than in the Sham group, while moscatilin administration rescued such decrements (Figure 6A–C). As illustrated in Figure 6D–G, DR mice exhibited prominently increased ROS and MDA levels and decreased SOD and CAT activities compared with sham mice. However, these changes were overturned by moscatilin administration, whereas DMSO had no such effect, confirming the antioxidant effect of moscatilin. Moreover, the increased secretion of VEGF, TNF‐α, IL‐1β, and IL‐6 in DR mouse models was inhibited by administration with moscatilin (Figure 6H–K), showing the anti‐inflammatory effect of moscatilin.

FIGURE 6Moscatilin attenuates oxidative stress and inflammation in Diabetic retinopathy (DR) mouse models. (A–C) Assessment of the development of Müller cells in mouse retina through dual‐immunofluorescence staining of GFAP and vimentin. Scale bar = 100 μm. (D) Determination of reactive oxygen species levels in the mouse retina through CM‐H_2_DCFDA staining. Scale bar = 200 μm. (E–G) Examination of MDA, SOD, and CAT levels in the retinas of mice mouse retina by commercial kits. (H–K) Detection of VEGF, TNF‐α, IL‐1β, and IL‐6 levels in the mouse retina using corresponding ELISA kits. *n* = 6/group. ***p < 0.001 versus Sham; ###*p* < 0.001 versus DR + DMSO. Values are expressed as the mean ± standard deviation of triplicate experiments. CAT, catalase; DMSO, dimethyl sulfoxide; ELISA, enzyme‐linked immunosorbent assay; GFAP, glial fibrillary acidic protein; IL‐1β, interleukin‐1β; IL‐6, interleukin‐6; MDA, malondialdehyde; SOD, superoxide dismutase; TNF‐α, tumor necrosis factor‐α; VEGF, vascular endothelial growth factor.

**Figure:**
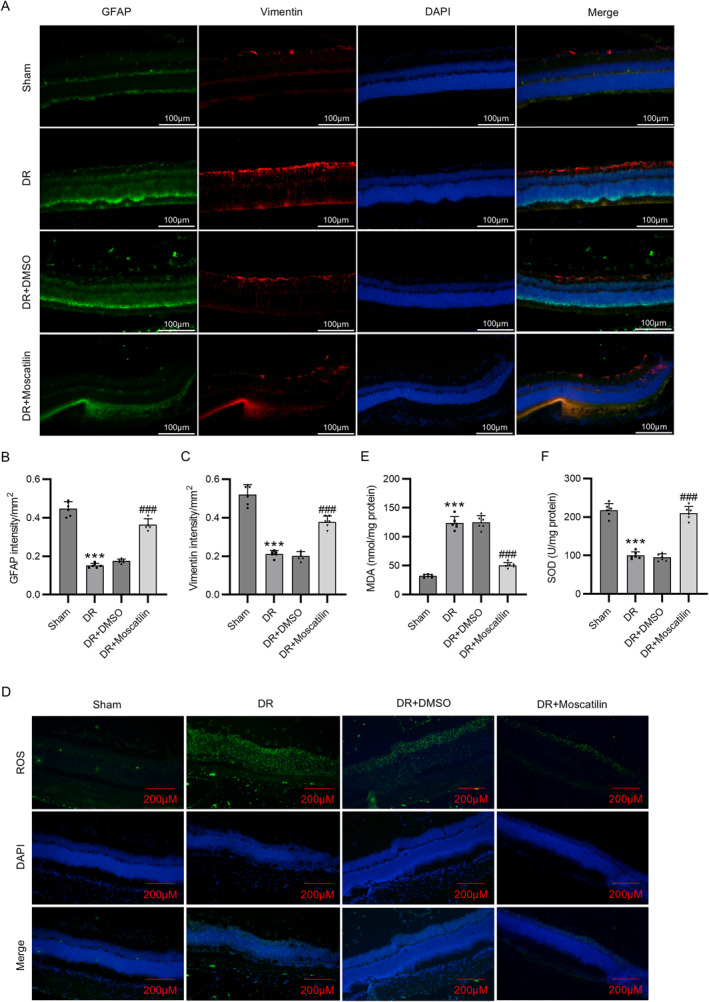

**Figure:**
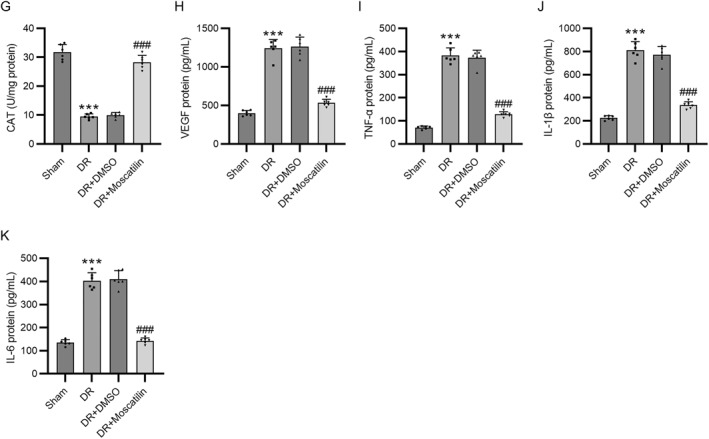

### 3.7 Moscatilin represses NF‐κB and p38/JNK pathway activation in DR mouse models

**FIGURE 7:**
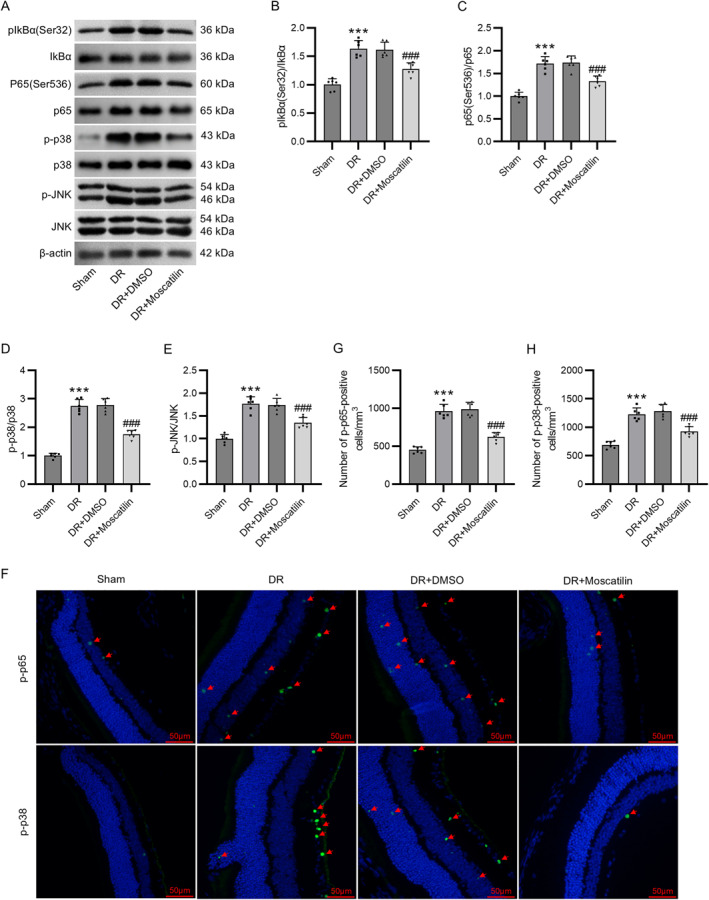
Moscatilin represses NF‐κB and p38/JNK pathway activation in Diabetic retinopathy (DR) mouse models. (A–E) Western blotting of p‐IkBα, IkBα, p‐p65, p65, p‐p38, p38, JNK, and p‐JNK protein levels in the mouse retina. (F–H) Assessment of p‐p65 and p‐p38 expression in the mouse retina through immunohistochemical staining. Scale bar = 50 μm. *n* = 6/group. ***p < 0.01 versus Sham; ###*p* < 0.01 versus DR + DMSO. Values are expressed as the mean ± standard deviation of triplicate experiments. DMSO, dimethyl sulfoxide; JNK, c‐Jun N‐terminal kinase; p‐IkBα, phosphorylated I‐kappa‐B‐alpha.

Finally, to investigate the molecular mechanism underlying the protective effects of moscatilin in vivo, the levels of molecules in the NF‐κB and p38/JNK pathways in the retinas of mice were tested. Through western blotting, we found that p‐IkBα, p‐p65, p‐p38, and p‐JNK protein levels were dramatically increased in the retinas of DR mice compared with sham mice. However, moscatilin administration reversed such an increase in their protein levels (Figure 7A–E). Immunofluorescence staining further validated that moscatilin treatment counteracted the enhancement in p‐p65 and p‐p38 expression in the retinas of DR mice (Figure 7F–H), indicating the inhibition of moscatilin on NF‐κB and p38/JNK pathway activation in DR mice.

## 4 DISCUSSION

Patients with a long history of DM tend to develop serious comorbidities, such as DR, which has increasing prevalence worldwide and is one prominent cause of visual impairment.^31^ The underlying pathogenesis of DR is complicated, and it is currently acknowledged that oxidative stress and inflammation are critical factors contributing to the occurrence and development of DR.^32^ Under the pathological stimuli, Müller cells, which are major macroglial cells that maintain the functions and metabolism of retinal neurons and blood vessels, are activated.^33^ In Müller cells, the balance between ROS and inflammatory cytokine production and elimination is disrupted in response to the constant stress of DR, which results in cell damage and worsens the disease.^34^ According to previous evidence, the use of natural agents with potent anti‐diabetic, anti‐oxidant, and anti‐inflammatory properties may be promising preventive and therapeutic methods for DR.^35^ In our study, we demonstrated that moscatilin alleviated oxidative stress and inflammation in both cellular and animal models of DR, which might depend on its inhibition on the p38/JNK and NF‐κB pathways.

Advanced glycation end products (AGEs) have been proven to play a crucial pathogenic role in the onset and progression of DR.^36^ Furthermore, the RAGE is widely expressed in various retinal cells and is upregulated in the retinas of diabetic patients, leading to the activation of pro‐oxidant and proinflammatory signaling pathways.^37^ This AGE‐RAGE axis appears to play a pivotal role in the sustained inflammation, neurodegeneration, and retinal microvascular dysfunction observed during DR.^38^, ^39^ Most recently, genipin was reported to ameliorate DR by targeting hypoxia‐inducible factor 1α and AGEs‐RAGE pathways.^40^ Furthermore, moscatilin exhibited a beneficial effect in ameliorating the pathophysiology of DM and its related complications by inhibiting RAGE signaling.^41^ In our study, the RAGE protein in Müller cells was markedly enhanced after HG stimulation, which however, was reduced by moscatilin pretreatment, suggesting the inhibitive effect of moscatilin on RAGE signaling in the development of DR.

At present, oxidative stress is considered as one component of DR pathogenesis.^42^ Oxidative stress represents excessive production of ROS and/or impaired scavenging mechanisms. The retina is rich in polyunsaturated fatty acids and has the greatest capacity for oxygen uptake and glucose oxidation compared to other tissues.^43^ During diabetes, this phenomenon makes the retina more susceptible to damage from oxidative stress.^44^ The hyperglycemic state can alter various biochemical pathways and enhance citric acid cycle activity, thereby leading to increased production of ROS in the retina.^45^ Multiple supplemented natural antioxidants, such as apocynin, green tea, quercetin, and cocoa have been revealed to be beneficial in animal models of DR since they inhibit endoplasmic reticulum stress and ROS production, decrease pro‐inflammatory cytokines and suppress apoptosis in the retina.^46^ Müller cells play an essential role in retinal antioxidant defense, and heme oxygenase‐1 (HO‐1) expression and ROS production in Müller cells can be remarkably elevated by HG.^47^ Besides, Müller cells are the primary cells that contain GSH, which is one major intracellular antioxidant molecule that acts directly through scavenging ROS and reactive nitrogen species and can be released in response to tissue stress to protect other cells from oxidant insults.^48^ Previously, moscatilin has been reported to exhibit its neuroprotective effect against Alzheimer's disease through attenuating oxidative stress.^49^ Herein, our data showed that HG‐induced elevation in ROS generation and intracellular MDA levels, and reduction in the GSH/GSSG ratio in Müller cells were inhibited by moscatilin pretreatment. Moreover, administration with moscatilin obviously decreased ROS and MDA levels and increased SOD and CAT levels in DR mice, which further confirmed the antioxidant role of moscatilin in DR. It has been well‐established that MAPKs, a family of protein kinases, affect cell development and ROS‐induced cell death. The antioxidants reduce MAPK activation and subsequently cellular ROS levels under oxidative stress conditions.^50^ Among the MAPK subfamily, extracellular signal‐regulated kinase 1/2 is responsible for modulating cellular growth and progression after being activated by mitogens and cell growth factors, while the p38 MAPK and JNK cascades are thought to be pro‐apoptotic cascades triggered in response to hypoxia or oxidative stress.^51^ Therefore, regulating the MAPK signaling cascade is crucial for protecting against ROS exposure in cells. Our findings illustrated that moscatilin treatment evidently reduced the phosphorylated level of p38 and JNK in HG‐exposed Müller cells and in DR mice, which endorses the protective role of moscatilin against oxidative stress in Müller cells and DR mice through inactivation of the p38/JNK pathway.

In addition, the development of DR is usually hallmarked by chronic low‐grade inflammatory response, which is related to the upregulation of various angiogenic factors (such as VEGF) and proinflammatory cytokines (such as TNF‐α, IL‐6, and IL‐1β) after the activation of microglia/macrophage in the retina.^52^ Compared to non‐diabetic people, enhanced levels of these particular factors can be observed in the aqueous humor of diabetic patients (with or without retinopathy).^53^ The inflammatory environment contributes to retinal damage during diabetes development, including neuronal and capillary degeneration, neovascularization, and blood‐retinal barrier breakdown.^54^ Müller cells are one of the major sources of proinflammatory cytokines in DR, which display a complex reactive phenotype in diabetes, with 33% of the differentially‐expressed genes being associated with inflammation.^55^ Coughlin et al. clarified that Müller cells released damaging pro‐inflammatory cytokines under high glucose conditions, contributing to chronic inflammation in diabetic retinas.^10^ NF‐κB, an important regulator of the inflammatory response, is increased by hyperglycemia in Müller cells as an early response and plays a vital role in inducing pro‐inflammatory cytokine production.^56^ Tu et al. elucidated that increased ROS production activated NF‐κB, which pathway was blocked through controlling oxidative stress in Müller cells.^16^ Several natural extracts such as erianin and asiatic acid have been confirmed to possess therapeutic benefits on DR by alleviating retinal inflammation through inhibiting the NF‐κB pathway.^57^, ^58^ Previously, moscatilin was reported to show effective anti‐inflammatory effects. For example, moscatilin mitigates liver injury in concanavalin A‐induced mouse models of autoimmune liver disease via inhibiting liver inflammation.^25^ Moscatilin inhibits the expression of proinflammatory enzymes cyclooxygenase 2 and inducible nitric oxide synthase in lipopolysaccharide‐stimulated murine macrophage‐derived RAW264.7 cells by suppressing NF‐κB nuclear translocation and activity.^59^ Herein, our data manifested that moscatilin treatment suppressed the upregulation in TNF‐α, IL‐1β, IL‐6, and VEGF levels in both HG‐stimulated Müller cells and DR mice. Moreover, moscatilin repressed the phosphorylation of IkBα and p65 protein levels, indicating that moscatilin might exert its anti‐inflammatory effects in DR through inactivating the NF‐κB signaling pathway.

**FIGURE 8:**
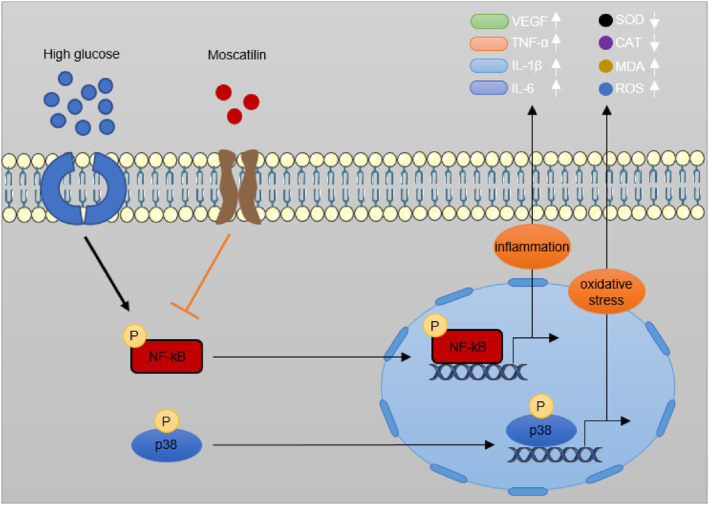
Schematic diagram showing that moscatilin protects against Diabetic retinopathy by alleviating oxidative stress and inflammation through inhibiting the activation of NF‐κB and p38/JNK pathways. JNK, c‐Jun N‐terminal kinase.

Collectively, our research demonstrated for the first time that moscatilin effectively ameliorated oxidative stress and inflammation in HG‐exposed Müller cells and in DR mice, and such a protective role was mostly dependent on the inhibition of p38 MAPK/JNK and NF‐κB signaling pathways (Figure 8). Our findings not only increase our understanding of the functions of moscatilin in regulating inflammation and oxidative stress in DR but also provide a potential candidate drug for DR treatment.

## AUTHOR CONTRIBUTION

Suhua Zhu and Man Zhang conceived and designed the experiments. Suhua Zhu, Man Zhang, Zhen Qu, Shengqiu Xu, Jie Peng and Fanjing Jiang carried out the experiments. Suhua Zhu, Man Zhang and Fanjing Jiang analyzed the data. Suhua Zhu, Man Zhang and Fanjing Jiang drafted the manuscript. All authors have read and approved the final manuscript.

## CONFLICT OF INTEREST STATEMENT

The authors declare that there are no competing interests in this study.

## ETHICS STATEMENT

The animal experiments were reviewed and approved by Institutional Ethics Review Committee of Xuzhou No.1 People's Hospital.

## Data Availability

The datasets used or analyzed during the current study are available from the corresponding author on reasonable request.
